# Mutations in *LRP5 *cause primary osteoporosis without features of OI by reducing Wnt signaling activity

**DOI:** 10.1186/1471-2350-13-26

**Published:** 2012-04-10

**Authors:** Johanna Korvala, Harald Jüppner, Outi Mäkitie, Etienne Sochett, Dirk Schnabel, Stefano Mora, Cynthia F Bartels, Matthew L Warman, Donald Deraska, William G Cole, Heini Hartikka, Leena Ala-Kokko, Minna Männikkö

**Affiliations:** 1 Oulu Center for Cell-Matrix Research, Biocenter and Department of Medical Biochemistry and Molecular Biology, University of Oulu, Oulu, Finland; 2 Departments of Medicine and Pediatrics, Massachusetts General Hospital and Harvard Medical School, Boston, MA, USA; 3 Children’s Hospital, Helsinki University Central Hospital and University of Helsinki, and Folkhälsan Research Center, Helsinki, Finland; 4 Hospital for Sick Children, University of Toronto, Toronto, ON, Canada; 5 Department for Pediatric Endocrinology and Diabetes, Otto-Heubner-Centrum für Kinder- und Jugendmedizin, Charite, University Medicine Berlin, Berlin, Germany; 6 Laboratory of Pediatric Endocrinology, BoNetwork, Division of Metabolic and Cardiovascular Sciences, San Raffaele Scientific Institute, Milan, Italy; 7 Case Western Reserve University, Department of Genetics, Cleveland, OH, USA; 8 Orthopaedic Research Laboratories, Children's Hospital Boston, Boston, MA, USA; 9 Department of Medicine, Winchester Hospital, Winchester, MA, USA; 10 Division of Orthopaedic Surgery, Hospital for Sick Children, University of Toronto, Toronto, ON, Canada; 11 Department of Surgery, North Karelia Central Hospital, Joensuu, Finland; 12 Connective Tissue Gene Tests, Allentown, PA, USA; 13 Department of Medical Biochemistry and Molecular Biology, University of Oulu, P.O. Box 5000, 90014 Oulu, Finland

## Abstract

**Background:**

Primary osteoporosis is a rare childhood-onset skeletal condition whose pathogenesis has been largely unknown. We have previously shown that primary osteoporosis can be caused by heterozygous missense mutations in the Low-density lipoprotein receptor-related protein 5 (*LRP5*) gene, and the role of *LRP5 *is further investigated here.

**Methods:**

*LRP5 *was analyzed in 18 otherwise healthy children and adolescents who had evidence of osteoporosis (manifested as reduced bone mineral density i.e. BMD, recurrent peripheral fractures and/or vertebral compression fractures) but who lacked the clinical features of osteogenesis imperfecta (OI) or other known syndromes linked to low BMD. Also 51 controls were analyzed. Methods used in the genetic analyses included direct sequencing and multiplex ligation-dependent probe amplification (MLPA). *In vitro *studies were performed using luciferase assay and quantitative real-time polymerase chain reaction (qPCR) to examine the effect of two novel and three previously identified mutations on the activity of canonical Wnt signaling and on expression of tryptophan hydroxylase 1 (*Tph1*) and 5-hydroxytryptamine (*5-Htr1b*).

**Results:**

Two novel *LRP5 *mutations (c.3446 T > A; p.L1149Q and c.3553 G > A; p.G1185R) were identified in two patients and their affected family members. *In vitro *analyses showed that one of these novel mutations together with two previously reported mutations (p.C913fs, p.R1036Q) significantly reduced the activity of the canonical Wnt signaling pathway. Such reductions may lead to decreased bone formation, and could explain the bone phenotype. Gut-derived Lrp5 has been shown to regulate serotonin synthesis by controlling the production of serotonin rate-limiting enzyme, Tph1. *LRP5 *mutations did not affect *Tph1 *expression, and only one mutant (p.L1149Q) reduced expression of serotonin receptor *5-Htr1b *(*p *< 0.002).

**Conclusions:**

Our results provide additional information on the role of *LRP5 *mutations and their effects on the development of juvenile-onset primary osteoporosis, and hence the pathogenesis of the disorder. The mutations causing primary osteoporosis reduce the signaling activity of the canonical Wnt signaling pathway and may therefore result in decreased bone formation. The specific mechanism affecting signaling activity remains to be resolved in future studies.

## Background

Idiopathic juvenile osteoporosis (IJO) without features of osteogenesis imperfecta (OI) is a rare bone condition that affects children and adolescents. It is thought to develop as the initiation and efficiency of bone remodeling becomes impaired, thus leading to a reduced quantity of cancellous bone [1]. The first symptoms of IJO appear well before puberty and the principal symptoms include reduced bone mineral density (BMD), vertebral compression fractures and metaphyseal fractures in the long bones. The fractures lead to bone pain and impaired mobility [1-3]. IJO is suggested to be inherited in an autosomal dominant manner [4]. Thus far only one gene, namely the gene encoding the low-density lipoprotein receptor-related protein 5 (*LRP5*), has been shown to cause juvenile-onset osteoporosis similar to IJO [4].

LRP5 has an essential role in the Wnt signaling pathway, since it acts as a co-receptor that binds Wnt proteins with Frizzled-receptors [5,6]. Mutations within the gene are known to lead to various bone disorders: gain-of-function mutations in the *LRP5 *gene can cause high-bone-mass (HBM) phenotypes in humans [7,8], whereas homozygous loss-of-function mutations cause osteoporosis-pseudoglioma syndrome (OPPG) characterized by early-onset osteoporosis and complications in eye development [9-11]. Similarly, transgenic mice with interrupted *Lrp5 *express a low bone mass phenotype, independent of Cbfa-1, including decreased osteoblast proliferation, osteopenia and persistent embryonic eye vascularization [12]. Furthermore, associations have also been reported between the *LRP5 *gene polymorphisms and bone mass and size [13-15].

LRP5 is widely expressed in most human tissues, with greater amounts in the liver and pancreas [16]. In bone, it is mainly expressed by the bone-forming cells, i.e. osteoblasts, in the endosteal and trabecular bone surfaces [7,9]. It is not known to be expressed by osteoclasts [9]. Recently, Lrp5 expressed in the murine duodenum was shown to affect the synthesis of gut-derived serotonin (5-hydroxytryptamine, i.e. 5-HT) by inhibiting expression of the serotonin rate-limiting enzyme tryptophan hydroxylase 1 (Tph1) [17]. Serotonin then affects bone formation, its effect being mediated by specific 5-HT transporters in the circulation and by binding to the 5-HT receptor 1 B (5-Htr1b) on osteoblasts [17,18]. However, other investigators have not observed a role for gut-expressed Lrp5 in regulating serotonin production or bone mass [19].

In the present study the role of *LRP5 *was explored further in 18 pediatric patients with primary osteoporosis without features of osteogenesis imperfecta (OI). *In vitro *cell culture studies were used to examine the effects of newly found mutations on LRP5 production, the activity of the Wnt signaling pathway, and the expression of *Tph1 *and *5-Htr1b*.

## Methods

### Subjects

The study included eight Italian and ten German patients. All 18 pediatric patients had been referred for recurrent fractures of long bones, bone pain, findings of osteopenia on imaging and/or low BMD. The diagnosis of primary osteoporosis was based on the following criteria: I) clinical exclusion of OI, II) exclusion of secondary causes of osteoporosis, and III) low BMD, defined as Z score below -2.0, history of recurrent peripheral fractures (≥3 fractures) caused by low impact trauma, and/or findings of vertebral compression fractures on x-ray films [19,20].

The control group, comprising 51 healthy individuals, was taken from the same geographical area as the two German patients who had novel *LRP5 *mutations. In addition, three affected and three healthy family members of the two probands with newly found mutations were analyzed. The study was approved by the local ethics committees, and signed informed consent was obtained from each subject.

### Molecular analysis

DNA was extracted from EDTA blood samples using standard procedures. The 23 exons and intronic boundaries of *LRP5 *were amplified using the polymerase chain reaction (PCR) method with AmpliTaq Gold DNA polymerase (Applied Biosystems), and dimethyl sulfoxide (DMSO) was added to the reaction mixture for exon 4. Exons 5 and 21 were amplified using AmpliTaq Gold360 (Applied Biosystems) and exon 1 was amplified with the GC Rich PCR kit (Roche Applied Sciences). PCR primer sequences are available on request. Mutation analysis was performed with direct sequencing using the ABI PRISM^® ^3100 Genetic Analyzer and BigDye terminator cycle sequencing chemistry (Applied Biosystems). GenBank accession number NG_015835.1 was used as a genomic *LRP5 *reference. Mutation nomenclature is in accordance with the guidelines by den Dunnen et al. [21], and the cDNA and protein reference sequences used were NM_002335.2 and NP_002326.2 (GenBank).

Samples were also screened for insertions or deletions using Multiplex Ligation-dependent Probe Amplification (MLPA) [22]. The MLPA analysis was performed according to MRC-Holland (Amsterdam, The Netherlands) procedure using *LRP5 *and control probes specifically designed in Dr. Warman's laboratory (Additional file 1: Table S1). The probes target sites in *LPR5 *differ from the sequence of a pseudogene containing *LRP5 *exons 3-9 (GenBank accession number AL022324). As a control for detecting deletion or duplication of the whole *LRP5 *gene (chromosome 11), a probe pair targeting for *acetylcholinesterase *(*ACHE*) exon 2 (chromosome 7) was used. Probes were synthesized by Integrated DNA Technologies (Coralville, IA). Unique amplicon sizes are presented in Additional file 2: Table S2. Amplification products were separated on ABI-PRISM^® ^3100 Genetic Analyzer (Applied Biosystems) in the Case Western Reserve Genomics Core Facility (Case Western Reserve University School of Medicine, Cleveland, Ohio), and results were analyzed with GeneScan^® ^Analysis 3.7 (Applied Biosystems). Peak heights of amplicons were normalized for the *ACHE *peak or for the *LRP5 *exon 15.

### *In vitro *studies

#### Constructs for in vitro studies

The wild type construct of full-length LRP5 with a carboxyl mycHis-tag (WT-LRP5-mycHis) was received from Dr. Warman's laboratory, having been created as described by Ai et al. [23]. The mutations identified in the osteoporosis patients were introduced into the WT-LRP5-mycHis construct using the QuikChange XL site-directed mutagenesis kit (Stratagene) and the resulting constructs were sequenced to confirm their correctness. Five LRP5 mutants were created that included two previously reported primary osteoporosis mutations (C913fs, R1036Q) [4], the two novel mutations (L1149Q, G1185R), and one HBM mutation G171V [13].

The reporter constructs SuperTOPflash (STF) and β-galactosidase (β-Gal-CMV) were received from Prof. Vainio's laboratory and were used to detect the activity of the canonical Wnt signaling pathway.

#### Expression of LRP5

Chinese Hamster Ovarian (CHO) cells were cultured with 10% fetal bovine serum (FBS) (HyClone) in Dulbecco's modified Eagle's medium (DMEM) (BIOCHROM AG), plated on 10 cm plates and transfected with 3 μg of WT-LRP5-mycHis or mutant construct using Lipofectamine transfection reagent (Invitrogen) according to the manufacturer's protocol. After 48 h of transfection, the cell medium was collected and the cells were lysed using 1% Triton-X-homogenisation buffer. A 7.5% SDS-PAGE gel was prepared and 25 μl of medium or cell lysate and 10 μl of SDS-PAGE loading buffer were loaded on the gel and analyzed under reducing conditions. Western blot analysis was performed using the Anti-Myc tag, clone 9E10 antibody (Upstate).

#### Producing Wnt3a -conditioned media

Wnt3a-conditioned medium was produced and collected from mouse L1 cells expressing Wnt3a (L Wnt3a; ATCC CRL-2647) according to the manufacturer's instructions. The control-conditioned medium (L1-CM) was prepared from a normal L1 cell line (ATCC CRL-2648) using the same protocol as for the L Wnt3a cells.

#### Luciferase gene reporter assay

##### LRP5 mutants and 10% FBS-DMEM medium

CHO cells were plated at 2 × 10^4 ^cells/well onto a 24-well plate and transfected 24 h later using Lipofectamine (Invitrogen). To study the effect of LRP5 mutants on Wnt signaling activity, the WT-LRP5 or mutant LRP5 construct (20 ng), STF (100 ng) and β-Gal-CMV (5 ng) were co-transfected into cells. The total amount of transfected DNA was adjusted to 250 ng/well by adding pcDNA3.1+ vector. Five hours later 10% FBS-DMEM was added to each well, and the cells were collected after a further 48 h. Each transfection was performed in triplicate and repeated at least three times on separate occasions.

##### LRP5 mutants and Wnt3a -conditioned medium

In experiments where Wnt3a was used to induce the activity of the pathway the transfections were performed as described above but with 300 μl of Wnt3a-CM or control L1-CM and 200 μl of 10% FBS-DMEM added to each well 5 h after transfection. The cells were collected 48 h after transfections.

##### Measurement of luciferase activity

Cell culture lysis reagent (CCLR) was used to lyse the cells according to the manufacturer's instructions (Promega). The luciferase (STF) activity was measured in a solution of 10 μl of cell lysate and 50 μl of Luciferase Assay Reagent (Promega) and the activity of β-galactosidase using 10 μl of cell lysate and 70 μl of 1 × CPRG substrate (chlorophenol red-β-D-galactopyranoside) according to the Stratagene instructions. In both cases a Victor_TM_^3 ^V 1420 Multilabel Counter (Perkin Elmer) was used, and the relative luciferase unit (RLU) was determined from the ratio between the luciferase and β-galactosidase activities.

### Quantitative real-time polymerase chain reaction (qPCR)

The effect of LRP5 mutants on the expression of *Tph1 *and *5-Htr1b *was studied by qPCR. CHO cells were plated at 1 × 10^5 ^cells/well on 6-well plates and transfected 24 h later using Lipofectamine (Invitrogen) with 2 μg of either WT-LRP5, the mutant LRP5 construct or pcDNA3.1+. Four hours later 10% FBS-DMEM or Wnt3a-CM or control L1-CM was added to each well (see detailed description of CM production above), and cells were collected 48 h after transfection. Each experiment was performed in triplicate and repeated at least three times on separate occasions.

Total RNA was isolated using the E.Z.N.A. RNA isolation kit including a treatment with RNase-free DNase (OMEGA Bio-Tek). RNA concentrations were measured using a NanoDrop™ ND-1000 spectrophotometer (Thermo Scientific) and cDNA was synthesized by reverse transcript PCR (RT-PCR) from 1 μg of extracted RNA using the iScript™ cDNA Synthesis kit (BioRad). Quantitative real-time PCR (qPCR) analyses of *Tph1 *and *5-Htr1b *expression were performed with specifically designed murine primers (available on request), and the *Tph1 *and *5-Htr1b *results were compared with those for a standard *β-actin *control. The qPCR reactions were performed using iTaq™ SYBR Green Supermix with ROX (BioRad) in a Mx3005P QPCR instrument (Stratagene), and the data were assessed using MxPRo - Mx3005P v4.10 software (Stratagene). The gene expression change upon treatment was presented as relative expression (fold relative to the non-treated control, i.e. pcDNA3.1+ transfected sample) after normalizing to *β-*actin, and was calculated by the 2^-ΔΔC^_T _method [24]. Student's *t *test was used to compare the expression of *Tph1 *and *5-Htr1b *in samples transfected with LRP5 mutants with expression in samples transfected with WT-LRP5.

### Statistical analysis

Statistical analyses were performed using Student's *t *test. Values of p < 0.05 were considered statistically significant. Results are presented as mean ± standard deviation (SD).

## Results

**Figure 1:**
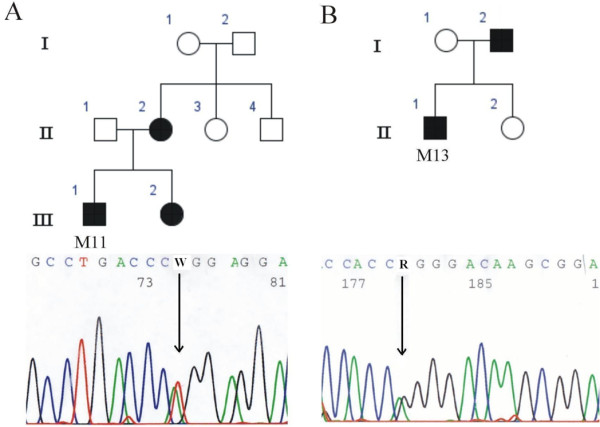
**A) The pedigree of patient M11, and a chromatogram showing the missense mutation c.3446 T > A (L1149Q), B) The pedigree of patient M13, and a chromatogram showing the missense mutation c.3553 G > A (G1185R)**. Affected individuals carrying the mutation are marked in black and the mutations are pointed out by means of arrows.

**Figure 2:**
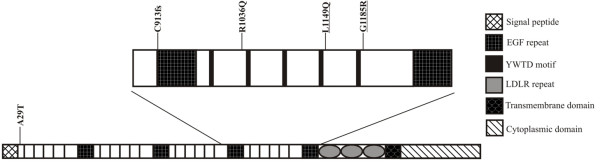
**Schematic presentation of the protein structure and domain organization of LRP5**. Mutations associated with primary osteoporosis are marked above the protein structure, and the two novel mutations found in the present study (L1149Q, G1185R) are underlined.

Altogether 18 patients were analyzed for *LRP5 *gene mutations by sequencing and MLPA. No changes were observed by MLPA (data not shown). Two new heterozygous missense mutations were found by sequencing, in patients M11 (Figure 1A III:1) and M13 (Figure 1B II:1). Both of these mutations, c.3446 T > A and c.3553 G > A, were located in exon 16 of the *LRP5 *gene, in the fourth YWTD/EGF domain of the LRP5 protein (Figure 2). The mutations led to L1149Q and G1185R amino acid substitutions, respectively. Changes were also detected in affected family members whereas neither was observed in the non-affected family members or in the control group.

Patient M11 (Figure 1A III:1) had recurrent fractures during childhood and adolescence and repeatedly subnormal BMD (the Z-score at lumbar spine at the time of diagnosis was -3.20), measured by dual-energy X-ray absorptiometry (DXA), and was diagnosed as having primary osteoporosis. M11 is currently 32 years and the most recent BMD measurements at the lumbar spine are normal (T-score 0.0) but osteopenic in the femoral neck (T-score -1.5). The patient has not received bisphosphonate treatment.

The mother (Figure 1A II:2) and sister (Figure 1A III:2) of patient M11 had the same mutation as the patient (c.3446 T > A; L1149Q), whereas the father (Figure 1A II:1) did not carry the mutation. In addition, the DNA samples of maternal grandparents (Figure 1A I:1 and I:2) and maternal aunt and uncle (Figure 1A II:3 and II:4) were analyzed, but none was found to have the mutation. Since the mother and sister have been diagnosed as having osteoporosis whereas the father shows no signs of reduced BMD, the genetic findings are concordant with the clinical status of the patient and his family members. The mother's lumbar spine and femoral neck BMD T-scores at age 48 years, after some years of bisphosphonate treatment, were -2.3 and -1.3. The sister's lumbar spine and femoral neck BMD T-scores at 25 years were -1.4 and -2.5.

For the rest of the relatives, the grandmother (Figure 1A I:1) had reduced BMD at age 83 years with lumbar spine and hip T-scores of -2.0 and -2.2, respectively. The grandfather's (Figure 1A I:2) BMD measurements at 83 years were somewhat reduced (BMD T-scores of -0.9 and -2.7 for lumbar spine and hip). The aunt (Figure 1A II:3) had osteopenia at 43 years (BMD T-scores 0.7 and -1.5 for lumbar spine and hip). No clinical data were available for the uncle of M11 (Figure 1A II:4). The results suggest that M11 inherited a *de novo *mutation that had arisen in his mother.

Patient M13 (Figure 1B II:1) was first presented at 13 years of age with multiple fractures in his shoulder, forearms and upper arms. The first fracture occurred at 7 years, and he has sustained altogether 8 fractures by age 14.5 years. When examined at the age of 13, the patient's alkaline phosphatase, parathyroid hormone, 25-hydroxyvitamin D_3_, calcium, phosphate and creatinine levels were normal and renal function was normal. Radiographs of the spine revealed reduced mineralization but no vertebral compression fractures or structural abnormalities. Peripheral quantitative computed tomography (pQCT; Stratec QCT 900) of the distal radius showed reduced total and trabecular volumetric BMD Z-scores of -1.5 and -2.8. The lumbar spine BMD Z-score, measured by DXA, was -2.4. The diagnosis of osteoporosis was confirmed by bone biopsy, which showed reduced bone volume and loss of trabecular connectivity, consistent with osteoporosis.

The father (Figure 1B I:2) of patient M13 (Figure 1B II:1) shared the mutation (c.3553 G > A; G1185R) with his son, but the change was not detected in the patient's unaffected mother (Figure 1B I:1) or sister (Figure 1B II:2). The father has slightly reduced BMD on DXA (T-score at lumbar spine -0.3 and at femoral neck -1.7). Otherwise the family has a negative history of osteoporosis.

Neither one of the novel mutations (L1149Q, G1185R) was detected in controls. The evolutionary importance of the observed disease-causing missense mutations was assessed by aligning the human protein sequences with the corresponding sequences of other species. Both the newly found mutations encode for conserved amino acids (Additional file 3: Figure S1).

### *In vitro *studies

Examination of the effect of the observed *LRP5 *gene mutations on protein expression using SDS-PAGE and Western blotting revealed no changes in expression levels (data not shown).

**Figure 3:**
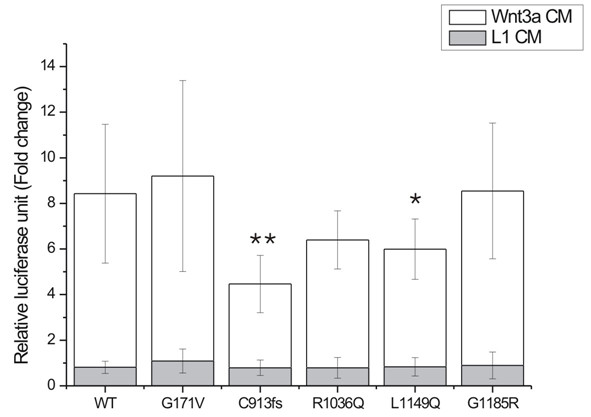
**Luciferase activity in CHO cells transfected with WT-LRP5 or mutant LRP5 in the presence of Wnt3a-CM (white bars) or L1 control medium (grey bars)**. The activities of the signaling pathway are presented in relative luciferase units (RLU) determined by the ratio between the luciferase and β-galactosidase activities and given as a fold change relative to corresponding samples treated with 10% FBS-DMEM. * *p *< 0.05, **p < 0.01 as compared with WT-LRP5. Results are expressed as mean ± SD.

The effect of the *LRP5 *mutations on the activity of the Wnt signaling pathway was studied by comparing the effect of the transfected mutant LRP5s with that of the WT-LRP5. These studies were performed in the presence of 10% FBS-DMEM, Wnt3a-CM or L1-CM, and the results were presented as fold changes relative to the results of 10% FBS-DMEM (Figure 3). As expected, Wnt3a-CM increased the activity of the pathway in all the LRP5 constructs, varying from 4 (C913fs) to 9-fold (G171V) as compared with the corresponding constructs when using 10% FBS-DMEM (Figure 3). The addition of Wnt3a-CM also brought out differences between the effects of the LRP5 constructs that were not seen in the samples treated with 10% FBS-DMEM or L1-CM. The HBM mutation G171V showed similar activity of the pathway to that of WT-LRP5, while a tendency for a decrease in activity was detected with mutations C913fs, R1036Q and L1149Q, the effects of C913fs and L1149Q being statistically significant (*p *= 0.0023 and *p *= 0.043, respectively) (Figure 3). The *LRP5 *mutant G1185R did not affect the signaling activity.

Expression levels of *Tph1 *and *5-Htr1b *were studied by qPCR in CHO cells transfected with WT-LRP5 or LRP5 mutants, and in the presence of either 10% FBS-DMEM, Wnt3a-CM or L1 control medium. *Tph1 *and *5-Htr1b *gene expressions were reported as fold changes relative to the untreated control sample (pcDNA3.1+) and normalized to *β-*actin. The expression resulting from each *LRP5 *mutant in the presence of Wnt3a-CM was first compared to the corresponding sample with L1-CM and then to expression with WT-LRP5 (using Wnt3a-CM).

**Figure 4:**
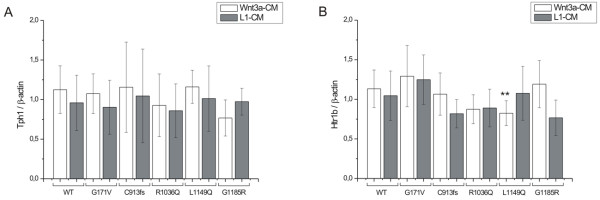
**A) *Tph1 *gene expression and B) 5-*Htr1b *gene expression in CHO cells transfected with wild type LRP5 (WT-LRP5) or mutant LRP5 and treated with Wnt3a-CM or L1-CM**. Expression of the *Tph1 *and 5-*Htr1b *genes is shown as a fold change relative to the untreated control (pcDNA3.1+) and normalized to *β-actin*. The effects of the LRP5 mutants on the expression of these genes were examined by comparing the results for the mutants with those for WT-LRP5 using Student's *t *test. The statistical significance of each pair is denoted below the mutant columns; **p < 0.01 as compared to WT-LRP5. Results are expressed as mean ± SD.

No differences were observed in the effect of the LRP5 mutants on *Tph1 *expression when using 10% FBS-DMEM (data not shown), and stimulating cells with Wnt3a-CM did not affect expression of *Tph1 *as compared with L1-CM (Figure 4A). Only one LRP5 mutant, G1185R, responded to Wnt3a-CM, although the difference relative to L1-CM was still marginal (*p *= 0.045).

The *5-Htr1b *expression was not affected by the LRP5 mutants in the presence of 10% FBS-DMEM (data not shown), but it did increase in cells transfected with LRP5 mutants C913fs and G1185R after the addition of Wnt3a-CM and as compared with L1-CM (*p *= 0.034 and *p *= 0.035, respectively; Figure 4B). These *5-Htr1b *expressions did not differ from that with WT-LRP5, however, while expression was significantly reduced in the LRP5 mutant L1149Q (*p *= 0.0051 as compared with WT-LRP5; Figure 4B). Also, the R1036Q mutation decreased *5-Htr1b *expression (*p *= 0.02), although the statistical significance of this effect was lost after two triplicate repeats (*p *= 0.50).

## Discussion

We have previously identified three mutations in the *LRP5 *gene that were associated with primary osteoporosis without features of OI [4]. The present work provides further proof of the role of *LRP5 *in the disorder by revealing two additional heterozygous missense mutations (L1149Q and G1185R) in patients with primary osteoporosis. Also, the *in vitro *studies showed that the *LRP5 *mutations C913fs and L1149Q alter Wnt signaling activity, as indicated by impaired activation of LRP5 by Wnt3a.

All *LRP5 *mutations associated with primary osteoporosis in our patient set (A29T, C913fs, R1036Q, L1149Q, G1185R) are located in the coding regions of the *LRP5 *gene. The L1149 and G1185 amino acids are conserved between species (Additional file 3: Figure S1), and are thereby likely to have structural and/or functional importance. Also, the site R1036 is quite well preserved as only one species out of eight differs from the human sequence at this position. The novel mutations (L1149Q and G1185R), as well as two mutations we have identified earlier (C913fs and R1036Q [4]), are located on the fourth propeller domain of LRP5 protein. Only one of the disease associated mutations (A29T) is situated in the first propeller domain of LRP5 [4]. However, primary osteoporosis and osteopenia have been confirmed in heterozygous carriers of OPPG-causing mutations located in other domains and on splice sites of *LRP5 *[10,25]. One of our patients (M13) with G1185R on the fourth propeller domain presented a graver phenotype than did his father who also had the mutation. The father had reduced BMD, but no osteoporosis. This finding is congruent with our previous and yet unpublished results ([4], Korvala et al. unpublished data) showing that phenotypes of affected offspring tend to be more severe than those of their parents. Reasons accounting for this may be variations in mutation penetrance or presence of other predisposing genetic factors [26,27] or the disorder may be multigenic in nature [28].

There is an interesting connection between the location of *LRP5 *mutations and resulting disorders (and presumably the disease causing mechanisms). HBM mutations are located in the first propeller domain of LRP5 whereas OPPG causing mutations are scattered mainly in the second and third propeller domains. Furthermore, different LRP5 domains bind to certain ligands in the Wnt signaling pathway: the first and second propeller domains of LRP5/6 participate in binding a certain class of the inducing ligands of the pathway e.g. Wnt1 and Wnt9b [29-31], but also the Wnt signaling inhibitors Wise, Sclerostin (SOST) and Dkk1 [31-33]. At the same time, the third and fourth propeller domains bind DKK1 [34,35] and another class of Wnt proteins e.g. Wnt3a (in LRP6) [30,31] while the cytoplasmic domain binds Axis inhibitor-1 (Axin) [36]. Moreover, the latest studies have indicated that different Wnts are able to bind to specific LRP6 propellers simultaneously [31], and compete with DKK1 binding [31,37]. These results may potentially be implicated also in LRP5. In conclusion, the site of mutation may be an important indicator for the resulting disorder, when assuming that the mutation affects the interaction between LRP5 and the ligand binding the mutation site.

Our *in vitro *studies with four *LRP5 *mutations causing primary osteoporosis showed that all the LRP5 constructs were able to mediate signaling and that the signaling activity was enhanced several-fold in all the constructs when Wnt3a was added (Figure 3), supporting the role of Wnt3a as a ligand for LRP5. Wnt3a also enabled us to elucidate the differences in signaling response between the LRP5 constructs: mutants C913fs and L1149Q reduced activity significantly (by 47% and 29%, respectively) as compared with WT-LRP5, and activity was also reduced by R1036Q (by 24%), whereas G1185R had no effect on signaling activity. Although no clinical significance has been reported for R1036Q (GenBank rs61889560), it has been detected in four OPPG patients [38] since its identification in a patient with primary osteoporosis [4], supporting its role in bone development or maintenance. Further functional studies are necessary for G1185R as no effect was detected using the current methods and a different experimental approach may identify the underlying mechanism.

The fact that the signaling activity of HBM mutation G171V in our study was close to that of WT-LRP5 is consistent with earlier findings that HBM-LRP5's are not constitutively active but need a Wnt ligand to be activated [39-41]. *In vitro *LRP5 studies by others have focused on HBM mutations and the few studies addressing the impact of mutations causing osteoporosis have mainly been associated with OPPG. These have shown that mutations causing OPPG reduce Wnt and/or Norrin signaling [11,38,42], while some mutants are trafficked unequally to the cell membrane [23]. Crabbe et al. [43] concluded that mutations associated with idiopathic osteoporosis in adult men may change the expression of LRP5 protein and/or interfere with the interaction of LRP5 with Mesd or with the Wnt/Fzd complex. Saarinen et al. [38] found an association between three homozygous OPPG mutations (R570W, R925C, R1036Q) and glucose tolerance, and suggested a potential association with diabetes. Taken together, our findings are in line with the results of other *in vitro *LRP5/OPPG studies showing that mutations associated with low bone mass disorders reduce the ability of LRP5 to mediate Wnt-induced signaling and consequently result in a low bone mass phenotype.

Since Yadav et al. [17] have shown that Lrp5 produced in the intestine can inhibit *Tph1 *expression, and consequently also 5-HT synthesis and bone formation, we examined whether the *LRP5 *mutations causing primary osteoporosis influence *Tph1 *and/or *5-Htr1b *expression in an *in vitro *system. Our results showed that only one of the mutations (L1149Q) reduced *5-Htr1b *expression significantly in the presence of Wnt3a (p < 0.002; Figure 4B), but neither HBM nor primary osteoporosis *LRP5 *mutations influenced *Tph1 *expression (Figure 4A). We cannot readily compare our *in vitro *results to the *in vivo *studies of Yadav et al. [17], and although the *5-Htr1b *finding is of potential interest, it is still tentative and further investigation using alternative methods is needed to examine its biological significance. One restriction of the current study is the use of only one reference gene, β-actin, which has been commonly used as a reference gene in human and murine gynecological tissue studies [44-47] and has shown stable expression in human endometrium [48].

The effect of 5-HT in regulating bone formation [17] still has some open questions as discussed by Warden et al. [18]. This is also illustrated by opposing results of Cui et al. [49] who showed that osteocyte specific activation or inactivation of *Lrp5 *in mice causes high or low bone mass, respectively [49]. Furthermore, the bone mass of these mice did not correlate with circulating serum serotonin levels nor did the bone markers or bone mass of ovariectomised mice change upon treatment with Tph1 inhibitor which still resulted in decreased circulating 5-HT [49]. Hence, the role of Wnt signaling pathway in bone cannot be totally overlooked. It is supported by both LRP5 studies [23,38,42], and by bone pathologies caused by mutations in other components of the pathway (e.g. SOST and DKK1). SOST mutations lead to severe HBM disorders, sclerosteosis and van Buchem disease [50,51], while DKK1 is shown to associate with bone lesions in multiple myeloma [52,53]. Taken together the studies describe the complexity of bone biology that we are only starting to understand and unravel.

## Conclusions

We have shown here that mutations causing juvenile-onset primary osteoporosis reduce the signaling activity of the canonical Wnt signaling pathway and may therefore result in decreased bone formation. Our preliminary results show reduced signaling in primary osteoporosis mutants but the specific mechanism affecting signaling activity remains to be resolved. Since the pathogenesis of primary osteoporosis has been largely unknown, our results provide additional information on the role of *LRP5 *mutations and their effects on the development of this disorder.

## Competing interests

The authors declare that they have no competing interests.

## Authors' contributions

JK and HH carried out the molecular genetic studies. JK performed the *in vitro *studies and drafted the manuscript. CFB and MLW performed the MLPA analysis and MLW provided the WT-LRP5 construct. HJ, OM, ES, DS, SM, DD, WGC, HH, LAK and MM conceived of the study, participated in its design and coordination and commented on the manuscript. All authors read and approved the manuscript.

## Pre-publication history

The pre-publication history for this paper can be accessed here:

http://www.biomedcentral.com/1471-2350/13/26/prepub

## Supplementary Material

Additional file 1 **Table S1**. Probes for MLPA of *LRP5 *and the gene control *Acetylcholinesterase *(*ACHE*). *LRP5 *probes were carefully designed not to overlap with a pseudogene (GenBank accession number AL022324) covering the exons 3-9 of *LRP5*. Click here for file

Additional file 2 **Table S2**. Expected amplicon sizes for *LRP5 *and *ACHE *(exon 2) in MLPA presented in increasing size order. Click here for file

Additional file 3 **Figure S1**. Partial alignment of the human LRP5 protein sequence with *Pan troglodyte*s, *Bos taurus Mus musculus, Rattus norvegicus, Gallus gallus, Danio rerio, Drosophila melanogaster *and *Anopheles gambiae*. The sites for three missense mutations associated with primary osteoporosis are shown within boxes. Lines show lacking sequence, and the arrowheads below the alignments point to sequence variations. Click here for file
