# Supplementary material for: Mutations in LRP5 cause primary osteoporosis without features of OI by reducing Wnt signaling activity
Source: BMC Med Genet. 2012 Apr 10;13:26. doi: 10.1186/1471-2350-13-26 (PMC3374890; doi:10.1186/1471-2350-13-26)
Supplement: Additional file 1 — Table S1. Probes for MLPA of LRP5 and the gene control Acetylcholinesterase (ACHE). LRP5 probes were carefully designed not to overlap with a pseudogene (GenBank accession number AL022324) covering the exons 3-9 of LRP5. [file 1471-2350-13-26-S1.DOC]

**Table 1**

Probes for MLPA of *LRP5* and the gene control *Acetylcholinesterase* (*ACHE*). *LRP5* probes were carefully designed not to overlap with a pseudogene (GenBank accession number AL022324) covering the exons 3-9 of *LRP5*.

| **Probe name** | **Probe length** | **Target** | **Sequence** |
| --- | --- | --- | --- |
| 1A | 43 bp | Intron 1 | **5’ GGGTTCCCTAAGGGTTGGA ccgccgtctcggaagcgacttggc** |
| 1B | 59 bp | Intron 1 | **5’ gagttgggagcgagttggggcgcg *tagatagcttcc* TCTAGATTGGATCTTGCTGGCAC** |
| 2A | 46 bp | Exon 2 | **5’ GGGTTCCCTAAGGGTTGGA ggatgcggccgcagtggacttccagtt** |
| 2B | 88 bp | Exon 2 | **5’ ttccaagggagccgtgtactggacagacgtgag *gtaggatttccaacttcccaaattgttcaagg***  **TCTAGATTGGATCTTGCTGGCAC** |
| 3A | 51 bp | Exon 3 | **5’ GGGTTCCCTAAGGGTTGGA cctggaggagcagaagctctactgggctgacg** |
| 3B | 91 bp | Exon 3 | **5’ ccaagctcagcttcatccaccgtgccaacc *ctatcctagtaggatttccaacttcccaaattgttcaa***  **TCTAGATTGGATCTTGCTGGCAC** |
| 4A | 56 bp | Exon 4 | **5’ GGGTTCCCTAAGGGTTGGA gggaagaggaaggagatcctgagtgccctctactcac** |
| 4B | 89 bp | Exon 4 | **5’ ccatggacatccaggtgctgagccaggag *gtaggatttccaacttcccaaattgttcaacaagatt***  **TCTAGATTGGATCTTGCTGGCAC** |
| 5E | 50 bp | Intron 4 | **5’ GGGTTCCCTAAGGGTTGGA cggggaggtccctgatgccacttgaggccga** |
| 5F | 67 bp | Intron 4 | **5’ tgtttgggcagagggacacactggaggctgtcacggg *ccgatgt* TCTAGATTGGATCTTGCTGGCAC** |
| 6A | 47 bp | Exon 6 | **5’ GGGTTCCCTAAGGGTTGGA gcacgccattgccatcgactacgacccg** |
| 6B | 103 bp | Exon 6 | **5’ ctagagggctatgtctactggacagatgacgaggtgcgg**  ***ctatcctattggtaggatttccaacttcccaaattgttcaa* TCTAGATTGGATCTTGCTGGCAC** |
| 7A | 57 bp | Exon 7 | **5’ GGGTTCCCTAAGGGTTGGA ggggagagaaccctaaaatcgagtgtgccaacttggat** |
| 7B | 75 bp | Exon 7 | **5’ gggcaggagcggcgtgtgctg *acaggatttccaactatcccaaattgttcaa***  **TCTAGATTGGATCTTGCTGGCAC** |
| 8A | 51 bp | Exon 8 | **5’ GGGTTCCCTAAGGGTTGGA ggggacttcatctactggactgactggcagcg** |
| 8B | 69 bp | Exon 8 | **5’ ccgcagcatcgagcgggtgcacaag *caatgttaacctaactctttg***  **TCTAGATTGGATCTTGCTGGCAC** |
| 9A | 42 bp | Exon 9 | **5’ GGGTTCCCTAAGGGTTGGA caacccggtgtggctgccccatc** |
| 9B | 70 bp | Exon 9 | **5’ ggcctggagctgctgagtgacatgaagacctg *tgatgttaccattag***  **TCTAGATTGGATCTTGCTGGCAC** |
| 10A | 47 bp | Exon 10 | **5’ GGGTTCCCTAAGGGTTGGA gcagttccggcaagtcctcgtgtggagg** |
| 10B | 63 bp | Exon 10 | **5’ gacttggacaacccgaggtcgctggcc *taatgttaccatt***  **TCTAGATTGGATCTTGCTGGCAC** |
| 11A | 52 bp | Exon 11 | **5’ GGGTTCCCTAAGGGTTGGA caccattgactacgctgaccagcgcctctactg** |
| 11B | 100 bp | Exon 11 | **5’ gaccgacctggacaccaacatgatcgagtcgtcc**  ***gtaggatttccaactcccaaattgttcaataggatttccaact* TCTAGATTGGATCTTGCTGGCAC** |
| 12A | 48 bp | Exon 12 | **5’ GGGTTCCCTAAGGGTTGGA cagcattgagcgggccgacaagactagcg** |
| 12B | 52 bp | Exon 12 | **5’ gccggaaccgcaccctcatccagg *acatg* TCTAGATTGGATCTTGCTGGCAC** |
| 13C | 55 bp | Exon 13 | **5’ GGGTTCCCTAAGGGTTGGA gacccactggacaagttcatctactgggtggatggg** |
| 13D | 53 bp | Exon 13 | **5’ cgccagaacatcaagcgagccaaggacgac TCTAGATTGGATCTTGCTGGCAC** |
| 14A | 46 bp | Exon 14 | **5’ GGGTTCCCTAAGGGTTGGA gcagccccacgacctcagcatcgacat** |
| 14B | 84 bp | Exon 14 | **5’ ctacagccggacactgttctggacgtgcgag *ttaggatttccaacttcccaaattgttcaa* TCTAGATTGGATCTTGCTGGCAC** |
| 15A | 50 bp | Exon 15 | **5’ GGGTTCCCTAAGGGTTGGA caacacactgggcaagctgttctgggtggac** |
| 15B | 87 bp | Exon 15 | **5’ gcggacctgaagcgcattgagagctgtgac *tcaagtaggatttccaacttcccaaattgttcaa***  **TCTAGATTGGATCTTGCTGGCAC** |
| 16A | 55 bp | Exon 16 | **5’ GGGTTCCCTAAGGGTTGGA gcctgaccatccttggcaagcatctctactggatcg** |
| 16B | 85 bp | Exon 16 | **5’ accgccagcagcagatgatcgagcgt *tcaattaggattatccaacttccacaaattgttcaa***  **TCTAGATTGGATCTTGCTGGCAC** |
| 17A | 50 bp | Exon 17 | **5’ GGGTTCCCTAAGGGTTGGA ggtggctgctcccacatctgtattgccaagg** |
| 17B | 72 bp | Exon 17 | **5’ gtgatgggacaccacggtgctcatgcc *gattaggattatccaacttcca* TCTAGATTGGATCTTGCTGGCAC** |
| 18A | 48 bp | Exon 18 | **5’ GGGTTCCCTAAGGGTTGGA ctgtgacggctttcccgagtgcgatgacc** |
| 18B | 57 bp | Exon 18 | **5’ agagcgacgaggagggctgccc *tagatagaccta* TCTAGATTGGATCTTGCTGGCAC** |
| 19A | 44 bp | Exon 19 | **5’ GGGTTCCCTAAGGGTTGGA cccaaccagttccggtgtgcgagcg** |
| 19B | 83 bp | Exon 19 | **5’ gccagtgtgtcctcatcaaacagcagtgcgactc *gtagttccaactccaaattgttcaag* TCTAGATTGGATCTTGCTGGCAC** |
| 20A | 43 bp | Exon 20 | **5’ GGGTTCCCTAAGGGTTGGA caagccgccctcagacgacagccc** |
| 20B | 52 bp | Exon 20 | **5’ ggcccacagcagtgccatcggg *atggaca* TCTAGATTGGATCTTGCTGGCAC** |
| 21A | 43 bp | Exon 21 | **5’ GGGTTCCCTAAGGGTTGGA ggcctcgtccagcagctcgtccag** |
| 21B | 54 bp | Exon 21 | **5’ cacgaaggccacgctgtacccgcc *tagatcc* TCTAGATTGGATCTTGCTGGCAC** |
| 22A | 50 bp | Intron 21 | **5’ GGGTTCCCTAAGGGTTGGA ggctctaagtcaccctggcttggctctcctc** |
| 22B | 75 bp | Intron 21 /  Exon 22 | **5’ agatcctgaacccgccgccctcc *gtaggatttccaacttcccaaattgttca* TCTAGATTGGATCTTGCTGGCAC** |
| 23A | 49 bp | Exon 23 | **5’ GGGTTCCCTAAGGGTTGGA cagccgctggaaggccagcaagtactacct** |
| 23B | 98 bp | Exon 23 | **5’ ggatttgaactcggactcagacccctatccacccc *gtaggatttccaacttcccaaattgttcatagcagatacc* TCTAGATTGGATCTTGCTGGCAC** |
| ACHE A | 45 bp | ACHE exon 2 | 5’ GGGTTCCCTAAGGGTTGGA gcgcaggtcctggtgaaccacgaatg |
| ACHE B | 70 bp | ACHE exon 2 | 5’ gcacgtgctgcctcaagaaagcgtcttc *ctaggatttccaacttccc* TCTAGATTGGATCTTGCTGGCAC |
| PCR F | 20 bp | Targets probes | 5’ FAM label-TGGGTTCCCTAAGGGTTGGA |
| PCR R | 23 bp | Targets probes | 5’ GTGCCAGCAAGATCCAATCTAGA |

Capital letters show common sequences that do not match genomic DNA, but to which PCR primers are targeted; Lower-case non-italicized letters show unique sequence that targets genomic DNA; Lower-case italicized letters indicate “stuffer” sequence used to make each exon’s amplicon size unique; All B probes were 5’ phosphorylated
