# Supplementary material for: Mutations in LRP5 cause primary osteoporosis without features of OI by reducing Wnt signaling activity
Source: BMC Med Genet. 2012 Apr 10;13:26. doi: 10.1186/1471-2350-13-26 (PMC3374890; doi:10.1186/1471-2350-13-26)
Supplement: Additional file 2 — Table S2. Expected amplicon sizes for LRP5 and ACHE (exon 2) in MLPA presented in increasing size order. [file 1471-2350-13-26-S2.DOC]

**Table 2**

Expected amplicon sizes for *LRP5* and *ACHE* (exon 2) in MLPA presented in increasing size order.

| **Exon target** | **Size*** |
| --- | --- |
| Exon 20 | 95 bp |
| Exon 21 | 97 bp |
| Exon 12 | 100 bp |
| Exon 1 | 102 bp |
| Exon 18 | 105 bp |
| Exon 13 | 108 bp |
| Exon 10 | 110 bp |
| Exon 9 | 112 bp |
| ACHE exon | 115 bp |
| Exon 5 | 117 bp |
| Exon 8 | 120 bp |
| Exon 17 | 122 bp |
| Exon 22 | 125 bp |
| Exon 19 | 127 bp |
| Exon 14 | 130 bp |
| Exon 7 | 132 bp |
| Exon 2 | 134 bp |
| Exon 15 | 137 bp |
| Exon 16 | 140 bp |
| Exon 3 | 142 bp |
| Exon 4 | 145 bp |
| Exon 23 | 147 bp |
| Exon 6 | 150 bp |
| Exon 11 | 152 bp |

*excluding overhanging as added by polymerase
