# Supplementary figures and images for: Mutations in LRP5 cause primary osteoporosis without features of OI by reducing Wnt signaling activity
Source: BMC Med Genet. 2012 Apr 10;13:26. doi: 10.1186/1471-2350-13-26 (PMC3374890; doi:10.1186/1471-2350-13-26)

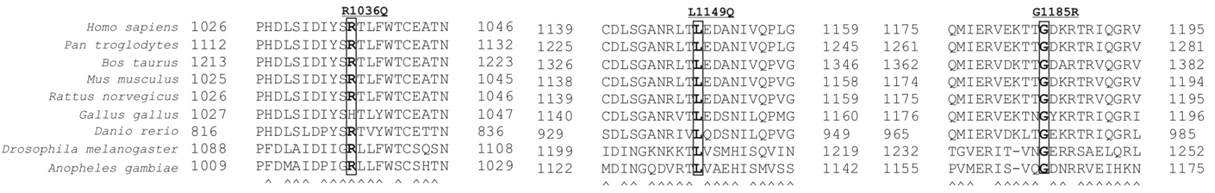

Supplement: Additional file 3 — Figure S1. Partial alignment of the human LRP5 protein sequence with Pan troglodytes, Bos taurus Mus musculus, Rattus norvegicus, Gallus gallus, Danio rerio, Drosophila melanogaster and Anopheles gambiae. The sites for three missense mutations associated with primary osteoporosis are shown within boxes. Lines show lacking sequence, and the arrowheads below the alignments point to sequence variations. [file 1471-2350-13-26-S3.TIFF]
